# First foods in a packaged world: Results from the COMMIT consortium to protect young child diets in Southeast Asia

**DOI:** 10.1111/mcn.13604

**Published:** 2023-12-13

**Authors:** Jessica L. Blankenship, Jessica M. White, Alissa Pries, Jane Badham, Arvind Betigeri, Janet Cade, Jennifer Cashin, Lucy Cosenza, Elizabeth Drummond, Anzélle Mulder, Nadine Nasser, Tuan Nguyen, Anusara Singhkumarwong, Lara Sweet, Diane Threpleton, Duong Vu, Elizabeth Zehner, Roland Kupka

**Affiliations:** ^1^ UNICEF East Asia and the Pacific Regional Office Bangkok Thailand; ^2^ Helen Keller International, New York New York USA; ^3^ JB Consultancy Johannesburg South Africa; ^4^ WFP Rome Italy; ^5^ School of Food Science and Nutrition University of Leeds Leeds UK; ^6^ Alive & Thrive Hanoi Vietnam; ^7^ Access to Nutrition Initiative (ATNI) Utrecht The Netherlands

**Keywords:** commercially produced complementary foods, labelling practices, legislation, nutrient composition, standards and guidelines

## Abstract

Although commercially produced complementary foods (CPCFs) are increasingly sold throughout Southeast Asia, concerns have been raised about CPCFs nutritional quality, labelling practices and the strength and scope of national CPCF regulations. The Consortium for Improving Complementary Foods in Southeast Asia (COMMIT), composed of UN agencies and civil society organizations, was formed to assess the nutrient gap in the diets of young children and the consumer, product and policy landscapes for CPCFs in seven Southeast Asian countries. Results from a nutrient gap assessment indicate that the diets of children aged 6–23 months are suboptimal and deficient in micronutrients. A consumer survey revealed that caretakers commonly use CPCFs, are conscious of the importance of nutrition and are influenced by label claims. Results from a CPCF benchmarking showed that many products sold in Southeast Asia contained added sugar or sweeteners, had a high total sugar and/or high sodium content and that no CPCF product adhered to all recommended labelling practices. Further, a legal review of national binding legal measures relevant to CPCFs showed minimal alignment with available global guidance. Urgent actions are necessary to strengthen national regulations related to CPCF nutrient composition and labelling practices. To speed progress, COMMIT developed a compendium of existing standards and global guidance to help countries align their national regulations with CPCF composition, labelling and production recommendations. Advocacy to garner public support for new or improved CPCF regulations, as well as strong government monitoring and enforcement of regulations, is crucial to support efforts to safeguard and improve the diets of older infants and young children in Southeast Asia.

## Key message

- Poor diet quality is a major driver of the triple burden of malnutrition in Southeast Asia, where food environments are rapidly changing and commercially produced complementary foods (CPCFs) are widely available.
- CPCFs are routinely purchased for children 6–23 months of age because of their convenience and perceived nutritional value. COMMIT (Consortium for Improving Complementary Foods in Southeast Asia) found that caregivers' purchasing decisions are strongly influenced by CPCF labelling claims related to naturalness and healthfulness.
- Many CPCFs sold are fortified with micronutrients; however, 62% of cereals and ready‐to‐eat foods and 85% of snack and finger foods did not meet global nutritional standards and no products fully conformed to global CPCF labelling standards, which include the prohibition of claims.
- CPCFs that do not adhere to recommended nutrient composition and labelling requirements are unsuitable for children 6–36 months and should be prohibited from promotion. Legal measures in the seven countries studied are insufficient and require urgent action. A compendium of existing CPCF standards and global guidance, developed by COMMIT, can be used as a resource to update and strengthen national legislation to safeguard older infants and young child nutrition.

## Graphical abstract

The COMMIT (Consortium for Improving Complementary Foods in Southeast Asia) assessments show that across Southeast Asia, the diets of older infants and young children (IYC) lack sufficient micronutrient content to adequately fuel their growth and development; that mothers of older IYC in urban centres purchase commercially produced complementary foods(CPCFs), look at and are conscious of their nutrient composition and are influenced by the information and claims on their labels; that there are critical gaps in the CPCF regulatory environment; and that many of the CPCF products currently on the market are of poor nutritional quality and/or use labelling practices that may mislead consumers.

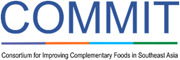

## 1 INTRODUCTION

Every child has the right to adequate food and nutrition (Convention on the Rights of the Child, 1989). Nevertheless, in Southeast Asia, over a quarter (26.4%) of children under 5 years of age are stunted, 7.8% are wasted (UNICEF, WHO, The World Bank, 2023) and more than half suffer from micronutrient deficiencies (Stevens et al., 2022; UNICEF, 2019). At the same time, the number of overweight children below 5 years of age has increased by half a million in the last decade (UNICEF, WHO, The World Bank, 2023). The poor quality of diets among children aged 6–23 months is one of the major drivers for this triple burden of malnutrition in Southeast Asia (Blankenship et al., 2020). Indeed, only half of children aged 6–23 months consume the minimum recommendation for a diverse diet, less than three‐quarters receive the recommended minimum meal frequency (UNICEF Division of Data, Analysis, Planning and Monitoring, 2022b) and only 59% of children aged 12–23 months are still breastfeeding (UNICEF Division of Data, Analysis, Planning and Monitoring, 2022a).

Despite economic progress in the region, Southeast Asian countries continue to grapple with persistent economic, physical and social barriers to improved nutrition and the socioeconomic costs of the coronavirus disease 2019 pandemic, while facing new and rapid changes in food environments (ASEAN, UNICEF, & WFP, 2022; UNICEF, 2021b). Present food environments in the region are characterized by an increasingly globalized and consolidated food system (Clapp, 2021), a growing presence of modern grocery retailers (GAIN JHU, 2020), rapid urbanization (United Nations, Department of Economic and Social Affairs, Population Division, 2018) and manufacturer's use of influential and pervasive marketing (Global Panel on Agriculture and Food Systems for Nutrition, 2016; UNICEF, 2020). These factors, and the powerful influencers of convenience, time and aspiration (Schmied et al., 2020), shape caregivers' decision‐making and behaviours for young child feeding (Development Initiatives, 2017). In many contexts, caregivers increasingly turn to commercially produced complementary foods (CPCFs), which are packaged foods marketed as suitable for older infants and young children (‘older IYC’) between the ages of 6 and 36 months as a feeding option (UNICEF, 2019, 2021a). The availability of CPCF is increasing rapidly throughout Southeast Asia. Market data indicate that in seven Southeast Asian countries, the CPCF market grew by 45% in the past 5 years, surpassing US$750 million in aggregate retail sales in 2022 (Euromonitor International, 2012–2022).

Despite their wide availability, the appropriateness of some CPCFs for older IYC has been brought into question in Southeast Asia. Research conducted in Cambodia, Indonesia and the Philippines found that most CPCF products contained added sugar and levels of sugar and/or sodium that exceeded recommended thresholds (Access to Nutrition Initiative, 2021; Bassetti et al., 2022). In Cambodia, CPCF products were labelled inappropriately, with all CPCF labels displaying compositional, nutrition and/or health claims, which global guidance discourages (Codex Alimentarius, 2004; WHO, 2017), and few included recommended messages on the protection and promotion of breastfeeding (Sweet et al., 2016).

These previous studies identified important concerns about CPCF nutritional quality and labelling that merited wider and more in‐depth study in the region. Furthermore, existing national legislation regulating CPCF composition and labelling has not been comprehensively evaluated in the Southeast Asia region. The Consortium for Improving Complementary Foods in Southeast Asia (COMMIT) was established to expand the evidence base on CPCF with the aim to improve the diets of older IYC through stronger CPCF regulation in seven countries in Southeast Asia (Cambodia, Lao People's Democratic Republic, Indonesia, Malaysia, Philippines, Thailand and Viet Nam). To inform actions at the national level, COMMIT conducted assessments to: (1) identify limiting micronutrients in diets during the complementary feeding period; (2) assess CPCF purchasing behaviours by urban mothers; (3) determine the alignment between mandatory national legislation relevant to CPCF and global guidance on CPCF nutrient composition and labelling practices; and (4) ascertain if CPCFs currently on the market meet global recommendations for nutrient composition and labelling practices (Table 1). These assessments were focused on CPCF promoted for older IYC, aged 6–36 months of age; however, both the analysis of limiting micronutrients in diets during the complementary feeding period and purchasing behaviours by urban mothers was focused on children 6–23 months of age due to data availability and higher expected consumption of CPCFs, respectively. COMMIT focused these assessments on the capital cities in each selected country due to the expected highest availability of CPCFs, as they are among the largest cities in the country, and because they house UNICEF national offices where national researchers could be engaged. Within the Southeast Asian region, Singapore and Brunei were excluded due to the absence of UNICEF operations in the country, Myanmar due to its ongoing national emergency and Timor Leste due to anticipated low market availability of CPCF.

**Table 1:** COMMIT activities across seven Southeast Asian countries.

COMMIT activity	Objective	Method	Supplement paper(s)
Activity 1	Identify limiting micronutrients in diets during the complementary feeding period	CONGA	Micronutrient gaps during the complementary feeding period in seven countries in Southeast Asia: A Comprehensive Nutrient Gap Assessment
Activity 2	Assess CPCF purchasing behaviours and motivating factor for urban mothers	Consumer perspective survey among mothers of older IYC	Health first, convenience second: Consumer perspectives on commercially produced complementary foods in five Southeast Asian capital cities
Activity 3	Determine alignment between national binding legal measures relevant to CPCF and global guidance on CPCF nutrient composition and labelling practices	Legal and policy review of relevant CPCF national binding legal measures	Widely consumed and under regulated: National binding legal measures related to commercially produced complementary foods in seven Southeast Asian countries are not fully aligned with global standards and guidelines
Activity 4	Ascertain if CPCF currently on the market meet global recommendations for nutrient composition and labelling practices	Benchmarking of nutrient composition and labelling practices of CPCF currently on the market against a nutrient profile model adapted for CPCF in Southeast Asia	Benchmarking the nutrient composition and labelling practices of dry or instant cereals for older infants and young children across seven Southeast Asian countries
			Benchmarking the nutrient composition and labelling practices of commercially produced ready‐to‐eat purées and meals for older infants and young children across seven Southeast Asian countries
			Benchmarking the nutrient composition and labelling practices of finger foods and snacks for infants and young children across seven Southeast Asian countries

Abbreviations: COMMIT, Consortium for Improving Complementary Foods in Southeast Asia; CONGA, Comprehensive Nutrient Gap Assessment.

The following six articles in this special issue of *Maternal & Child Nutrition* present the findings of these COMMIT assessments and present recommendations on how countries can strengthen their regulatory environments for CPCF through a holistic national strategy.

## 2 SUMMARY OF EVIDENCE PRESENTED IN THIS SPECIAL ISSUE

### 2.1 Objective 1: Identify limiting micronutrients in diets during the complementary feeding period

Poor dietary diversity and inadequate consumption of nutrient‐rich foods during the complementary feeding period can increase the risk of micronutrient deficiencies among older IYC and may impair their physical and cognitive development (Aguayo et al., 2016; Prado & Dewey, 2014; UNICEF, 2016). Global indicators for assessing IYC feeding practices are limited in that they cannot specify the magnitude or significance of specific micronutrient gaps in the diet (UNICEF, WHO, FANTA/FHI 360, & USAID, 2017; WHO & UNICEF, 2018). Although anaemia is one well‐documented public health concern with relevance to micronutrient malnutrition (ASEAN, UNICEF, & WFP, 2022), data on specific micronutrient gaps in child diets is often unavailable, underused or misinterpreted, thus limiting governments' ability to design effective policies and programmes to address these gaps (Beal et al., 2021; White et al., 2021).

In this issue, White et al. (2023) present the results of a Comprehensive Nutrient Gap Assessment (CONGA) of diets during the complementary feeding period in the seven Southeast Asia COMMIT countries. The CONGA aimed to estimate gaps of 12 micronutrients commonly lacking in the diets of older IYC and establish the certainty of evidence available to examine these gaps. The authors identified gaps in iron, vitamin D, zinc, and a potential gap in calcium, during the complementary feeding period and offered suggestions on how to address these gaps. To address the limited availability of existing data, the authors also call for new data collection and evidence generation on micronutrient availability, intake and deficiency across Southeast Asia.

### 2.2 Objective 2: Assess CPCF purchasing behaviours and motivating factors for urban mothers

Despite tremendous growth in the market availability and sale of CPCF products across the Southeast Asia region, caregivers' perceptions and practices related to CPCF are not well understood. In a previous study conducted in 18 countries including the Philippines and Indonesia in Southeast Asia, mothers said they fed CPCF to their children because the products were convenient, readily available and requested by their children (Schmied et al., 2020).

In this issue, Walls et al. (2023) present findings from online surveys conducted among mothers of children aged 6–23 months living in Bangkok, Thailand; Hanoi, Viet Nam; Jakarta, Indonesia; Kuala Lumpur, Malaysia; and Manila, Philippines. The aim of this survey was to explore purchasing behaviours and motivating factors for purchasing CPCF. The authors found that among mothers surveyed, who were primarily of middle or high socioeconomic status, almost all reported purchasing CPCF for their older IYC and that most provided CPCF to children aged 6–23 months at least once a day. While the convenience of these products was important to caregivers, the perceived nutritional value of the products was the primary motivation for CPCF purchases in all five capital cities. Mothers indicated that they reviewed nutrition information on CPCF product labels and were influenced by the information and claims made on the labels. Claims, such as ‘natural’, on CPCF labels strongly influenced the purchasing decisions of mothers.

### 2.3 Objective 3: Determine alignment between national mandatory legislation relevant to CPCF and global guidance

To inform the development of national legislation on CPCF nutrient composition and labelling, the WHO published *Guidance on Ending the Inappropriate Promotion of Foods for Infants and Young Children* (WHO Guidance) (WHO, 2016b) and Codex Alimentarius (Codex) developed six relevant standards and guidelines (Codex Alimentarius, 1981, 2004, 2006, 2011, 2013, 2018).

However, the Codex Standards and Guidelines for CPCF have been recognized by the World Health Assembly (WHO, 2016a) as inadequate because of their infrequent updating, limited scope of nutrient composition requirements and lack of labelling practice requirements to sufficiently protect and promote breastfeeding. In response, the WHO Regional Office for Europe (WHO Europe) developed a nutrient profile model (NPM) for CPCF (WHO Regional Office for Europe, 2019, 2022). This NPM is the first guidance document that provides recommendations for both nutrient composition and labelling for a range of CPCF product categories. It intends to guide CPCF policy reform in support of improved nutrition among older IYC.

In this issue, Blankenship et al. (2023) reviewed mandatory national policies, standards and legislation (binding legal measures) relevant to CPCF in the seven COMMIT countries and compared their alignment to the six relevant Codex Standards and Guidelines (Codex Alimentarius, 1981, 2004, 2006, 2011, 2013, 2018), the WHO Guidance (WHO, 2017) and the nutrient composition and labelling requirements in an adapted version of the WHO Europe NPM for CPCF. In the first analysis of this kind globally, the authors found that no country was fully aligned with the available global guidance. Alignment with Codex Standards and Guidelines specific to CPCF nutrient composition and labelling was limited, with only two countries in full alignment with at least one of these Standards and/or Guidelines, and three countries with no national legal measures that incorporated any element of these Codex documents. Only one country was in full alignment with the WHO Guidance and no country was fully aligned with the adapted version of the WHO Europe NPM for CPCF. The findings from Blankenship et al. (2023) indicate that existing legal measures in the COMMIT countries in Southeast Asia are insufficient to ensure CPCF are nutritionally adequate and appropriately labelled.

### 2.4 Objective 4: Ascertain if CPCF currently on the market meets recommended requirements for nutrient composition and labelling practices

To fill the evidence gap on the nutritional suitability and appropriateness of labelling practices among CPCF available in the Southeast Asia region, COMMIT benchmarked CPCF products purchased in the capital cities of the seven COMMIT countries against the nutrient composition and labelling requirements in the adapted version of the WHO Europe NPM for CPCF. A total of 1635 CPCF products were identified; of these, three CPCF product categories comprised 95% of the market: snacks or finger foods (37%), CPCF cereals (30%) and ready‐to‐eat purées (28%). Given product‐related specificities in nutrient composition and labelling requirements, these product groups were assessed separately. Further, these product categories were presented separately in three papers due to their distinct function and use for feeding of older IYC. Dry or instant cereals are often the first CPCF products introduced into diets among this age group, and if appropriately formulated and labelled, can serve as a vehicle for vital vitamins and minerals. Conversely, CPCF finger foods and snacks serve as a convenience food for caregivers and often mimic snack foods consumed by older population groups, typically containing added sugars and excessive levels of total sugar/sodium. Ready‐to‐eat purées and meals are sometimes used as meals but sometimes also as convenience foods and the composition of these products varies in appropriateness—some can introduce older IYC to new fruits and vegetables; however, some can be excessively sweet. Detailing the nutrient profiling performance of these three distinct CPCF product categories across these papers allows these issues and concerns to be explored and discussed fully. Products were required to pass both the nutrient composition and labelling requirements to be considered suitable for promotion for older IYC.

Three articles in this issue present the results of the assessments on specific CPCF product types. Bassetti et al. (2023) and Pries et al. (2023) found that over a third of CPCF cereals (38%) and ready‐to‐eat purées (38%) met all required nutrient composition requirements in the adapted WHO Europe NPM for CPCF, compared to 15% of CPCF snacks and finger foods. The presence of added sugar/sweeteners, high total sugar content and/or high sodium content were the most common reasons why CPCF products failed the nutrient composition assessment of the adapted WHO Europe NPM for CPCF. Among CPCF snacks and finger foods, the median total sugar and sodium content per 100 kcal of product was 3.0 g and 17 mg, respectively. However, none of the 1635 CPCF products assessed passed on all the labelling requirements of the adapted WHO Europe NPM for CPCF. Nearly all CPCF products contained prohibited compositional claims intended to influence caregivers as to the ‘healthfulness’ of the product. CPCF labels frequently included claims declaring the foods as ‘natural’, ‘wholesome’ and ‘healthful’. While the majority of CPCF products (95%) did not suggest superiority or equivalence to breast milk, over 25% of all CPCF products were marketed as suitable for infants under 6 months of age or lacked a minimum recommended age of introduction of at least 6 months, inappropriate practices that can negatively impact breastfeeding practices.

In the assessment of CPCF cereals, Bassetti et al. (2023) additionally analysed the presence and appropriateness of fortification with essential micronutrients. Over two‐thirds of CPCF cereals (69.2%) were fortified with essential micronutrients despite a lack of mandatory fortification for CPCF cereals in four of the seven COMMIT countries. Further, most fortified CPCF cereals were appropriately fortified and met the recommended fortification levels suggested in the Codex Alimentarius Guidelines for Formulated Complementary Foods (Codex Alimentarius, 2013).

## 3 WAY FORWARD

This special issue presents findings from four assessments that aim to provide evidence to inform and strengthen the regulatory environment for CPCF in Southeast Asia. These assessments show that across the region, the diets of older IYC lack sufficient micronutrient content to adequately fuel their growth and development; that mothers of older IYC in urban centres purchase CPCF, look at and are conscious of their nutrient composition and are influenced by the information and claims on their labels; that there are critical gaps in the CPCF regulatory environment; and that many of the CPCF products currently on the market are of poor nutritional quality and/or use labelling practices that may mislead consumers.

Given these findings, the regulation of CPCF needs to be strengthened. Global guidance documents exist to inform the design of national binding legal CPCF measures (Table 2). However, relevant information is spread across 17 documents, limiting the usability and uptake of the global guidance documents at the national level. To address this challenge, COMMIT developed a single compendium of existing standards and global guidance that outlines the minimal essential nutrient composition, production and labelling requirements recommended for adoption into national binding legal measures regulating CPCF (https://www.unicef.org/eap/media/14941/file). It is a comprehensive document inclusive of existing Codex Standards and Guidelines, Commission of the European Communities Commission Directive Standard for CPCF and WHO Guidance, adapted to incorporate mandatory fortification of older IYC cereals with iron, zinc and calcium to address the specific micronutrient gap requirements in the Southeast Asian context. Countries are encouraged to use this compendium as a tool for updating or developing new national CPCF‐binding legal measures.

**Table 2:** List of international guidelines and standards used as reference documents in the compendium of international standards and guidelines for the improved composition and labelling of commercially produced complementary foods in Southeast Asia.

Publishing body	Title
Codex	Standard for Processed Cereal‐based Foods for Infants and Young Children, CODEX STAN 74‐1981
Codex	Standard for Canned Baby Foods, CODEX STAN 73‐1981
Codex	Guidelines on Formulated Complementary Foods for Older Infants and Young Children, CAC/GL 8‐1991
Codex	General Standard for The Labelling of Pre‐packaged Foods, CODEX STAN 1‐1985
Codex	Guidelines on Nutrition Labelling, CAC/GL 2‐1985
Codex	Guidelines for Use of Nutrition and Health Claims, CAC/GL 23‐1997
Codex	Advisory Lists of Nutrient Compounds for Use in Foods for Special Dietary Uses Intended for Infants and Children, CAC/GL 10‐1979
Codex	General Standard for Food Additives Codex, STAN 192‐1995
Codex	General Principles of Food Hygiene, CAC/RCP 1‐1969
Codex	Code of Hygienic Practice for Powdered Formulae for Infants and Young Children, CAC/RCP 66‐2008
Codex	Principles for the establishment and application of microbiological criteria for foods, CAC/GL 21‐1997
Codex	General principles for the addition of essential nutrients to foods, CAC/GL 9‐1987
Codex	Standard for infant formula and formulas for special medical purposes intended for infants, Codex STAN 72‐1981
Codex	Recommended methods of analysis and sampling, CODEX STAN 234‐1999
Commission of the European Communities	European Commission Directive 2006/125/EC on processed cereal‐based foods and baby foods for infants and young children
WHO Regional Office for Europe	Nutrient and promotion profile model: supporting appropriate promotion of food products for infants and young children 6–36 months in the WHO European Region
COMMIT initiative	COMMIT Initiative Recommendations for Nutrient Composition and Labelling Practice Requirements of commercially produced complementary foods

Abbreviations: COMMIT, Consortium for Improving Complementary Foods in Southeast Asia; WHO, World Health Organization.

Advocacy from civil society organizations and the public can be an effective mechanism to garner support to update or develop new national binding CPCF legal measures. In some settings, consumer‐facing campaigns can build awareness of the poor nutritional quality of CPCF and create demand for more effective regulation of CPCF products. Recent campaigns in the United Kingdom have also spotlighted the concerning levels of free and added sugars in CPCF products, as well as misleading health claims found on labels (Crawley & Westland, 2017). Building awareness of these issues among caregivers can trigger demand for stronger regulation of CPCF products, and findings from market surveys and product assessments can also provide governments with the evidence needed to spur policy action.

The implementation of CPCF standards is dependent on government monitoring and enforcement of these legal measures. Experiences from implementation of the International Code of Marketing of Breast‐milk Substitutes and subsequent relevant World Health Assembly resolutions (the ‘Code’) can provide useful lessons on the challenges for implementation, monitoring and enforcement of CPCF regulations (WHO et al., 2016c). A 2021 analysis from five countries in Southeast Asia recommended that for more effective Code implementation, the roles and responsibilities of relevant agencies need to be clarified, capacities of key technical enforcement staff need to be enhanced and Code monitoring needs to be integrated and budgeted for through existing government systems (UNICEF EAPRO, 2021). The need for effective integration with government systems, such as food control, has also been highlighted by a review of large‐scale food fortifications worldwide (Mkambula et al., 2020). These lessons are crucial for the effective implementation of CPCF legal measures.

## 4 CONCLUSION

The market for CPCF in Southeast Asia is growing (Euromonitor International, 2012–2022). At the same time, existing CPCF‐related legislation is insufficient to protect optimal older IYC feeding. Urgent actions are therefore needed to better regulate CPCF at the national level. The COMMIT Compendium is a tool that can be used by countries to facilitate this. COMMIT is the most comprehensive effort to date documenting the regulation, use and quality of CPCF and the first to develop recommendations to strengthen the regulatory environment for CPCF. On the basis of this work, we conclude that any CPCF product that does not adhere to recommended nutrient composition and labelling requirements be prohibited from promotion, that is, sold as suitable for children 6–36 months of age. The COMMIT partners hope that the work documented in this special issue of MCN will help to safeguard against the current risks associated with CPCF use and support efforts to address the triple burden of malnutrition in Southeast Asia.

## AUTHOR CONTRIBUTIONS

Jessica L. Blankenship and Roland Kupka are UNICEF staff members and Jessica M. White is a UNICEF consultant. The opinions and statements in this article are those of the authors and may not reflect official UNICEF policies.

## CONFLICT OF INTEREST STATEMENT

The authors declare no conflict of interest.

## Data Availability

The data that support the findings of this study are available from the corresponding author upon reasonable request.
